# Multiomics profiling Identifies MCMBP as a prognostic biomarker and a potential immune-related target in pancreatic ductal adenocarcinoma via the JAK–STAT3 pathway

**DOI:** 10.3389/fimmu.2025.1621927

**Published:** 2025-11-19

**Authors:** Xinghai Zhang, Jiaqi Ma, Hao Yuan, Yuzhuo Li, Yixin Wang, Yujia Chen, Lei Zhang, Fangxuan Li, Xi Ma, Bixuan Li, Wen Xu, Yang Wang

**Affiliations:** 1 Department of Xi’an Key Laboratory of Pathogenic Microorganism and Tumor Immunity Faculty of Basic Medicine, Xi’an Medical University, Xi’an, China; 2 Department of Pathogen Biology of Basic Medicine, Xi’an Medical University, Xi’an, China

**Keywords:** pancreatic ductal adenocarcinoma, MCMBP, immunotherapy, prognosis, JAK-STAT3 pathway

## Abstract

**Background:**

Microchromosome maintenance protein-binding protein (MCMBP) is aberrantly expressed in cancers and proposed as a diagnostic marker and therapeutic target, but its role in pancreatic ductal adenocarcinoma (PAAD) remains unclear.

**Methods:**

We performed a comprehensive analysis of MCMBP in PAAD using multi-omics data resources, including TCGA, GTEx, CPTAC, GEO, GDSC, TIDE, HPA, MethSurv, DiseaseMeth, and LinkedOmicsKB. We examined its prognostic characteristics, epigenetic alterations, immune infiltration, immunotherapy response, and drug sensitivity. By integrating transcriptomic, proteomic, and phosphoproteomic data, we explored the biological functions and pathways of MCMBP. Sensitive drugs related to MCMBP were identified through the GDSC and Connectivity Map (CMap) drug libraries, with further functional insights obtained through GO and KEGG enrichment analyses. Potential mechanisms were investigated via gene functional experiments, phos-phorylation site predictions from LinkedOmicsKB, and protein expression validation.

**Results:**

Pan-cancer analysis revealed that MCMBP overexpression correlates with poor prognosis, including in PAAD. Cox regression identified MCMBP as an independent prognostic factor for PAAD. Low DNA methylation and high m6A modification of MCMBP may promote PAAD progression and correlate with adverse prognosis. Ge-ne function and immune infiltration analyses indicated that high MCMBP expression is closely associated with immune-related pathways, tumor cell proliferation, survival, and immune cell differentiation, and may promote Treg accumulation and immune ch-eckpoint upregulation. PAAD patients with low MCMBP expression exhibited greate-r sensitivity to anti-PD-L1 immunotherapy, suggesting a potential synergistic effect o-f MCMBP expression with anti-PD-L1 treatment. High MCMBP expression was ass-ociated with sensitivity to Gemcitabine combined with Paclitaxel, as well as small mo-lecules such as Tozasertib and Motesanib. MCMBP knockdown inhibited PAAD cell proliferation, migration, invasion, and G1-S transition. Immunohistochemical results s-howed that high MCMBP expression correlated with elevated PD-L1 levels and redu-ced CD4+ T cell infiltration in PAAD, which significantly associated with poor prog-nosis. MCMBP modulated PD-L1 through activation of the JAK-STAT3 signaling pat-hway, thereby promoting PAAD progression.

**Conclusions:**

Overexpression of MCMBP may serve as a prognostic biomarker and p-otential therapeutic target in PAAD. It drives PAAD progression by activating the JAK-STAT3 pathway to upregulate PD-L1.

## Introduction

Pancreatic ductal adenocarcinoma is a highly lethal malignancy characterized by a lack of early detection methods, resulting in most patients being diagnosed at advanced stages and experiencing poor surgical outcomes (1, 2). Neoadjuvant chemotherapy based on the FOLFIRINOX regimen is frequently used, however, its efficacy is often constrained by chemotherapy resistance, driven by genomic instability and tumor micro-environment (TME) heterogeneity (3, 4). Anti-PD-L1 therapy, which acts by modulating the immune microenvironment and enhancing T-cell-mediated antitumor activity, represents a promising strategy to overcome chemoresistance (5). Nevertheless, in PAAD, widespread hypoxia exacerbates DNA replication stress, triggers inflammatory factor release, and promotes the recruitment and functional enhancement of regulatory T cells (Tregs), collectively fostering a profoundly immunosuppressive TME (6–8). Consequently, anti-PD-L1 monotherapy is often insufficient to counteract this immunosuppression. Therefore, identifying TME-related prognostic biomarkers for combination with anti-PD-L1 therapy presents a potential approach to improve treatment outcomes.

MCMBP is a molecular chaperone that facilitates the assembly of the minichromosome maintenance (MCM) complex through its nuclear localization signal (NLS) and WalkerB-like motif, thereby preventing its cytoplasmic degradation and ensuring accurate DNA replication and cell cycle progression (9). It also interacts with MCM3 and MCM5 subunits to establish backup replication origins, contributing to genomic stability (10). Studies suggest that MCMBP-mediated dysregulation of replication stress in malignant cells may increase their susceptibility to certain therapies, highlighting its potential as an anticancer target (9). In hepatocellular carcinoma, MCMBP promotes tumor progression by regulating DNA replication and the cell cycle (11). In breast cancer, high MCMBP expression is associated with poor survival and correlates with estrogen receptor (ER)-negative status, underscoring its prognostic relevance (12). Similarly, in colorectal cancer, elevated MCMBP expression is linked to increased recurrence risk, suggesting its utility as a diagnostic biomarker (12). Moreover, MCMBP is highly expressed in proliferating B cells, implying a potential role in immune regulation (12). Despite these insights, the role of MCMBP in PAAD remains largely unexplored.

**Figure 1:**
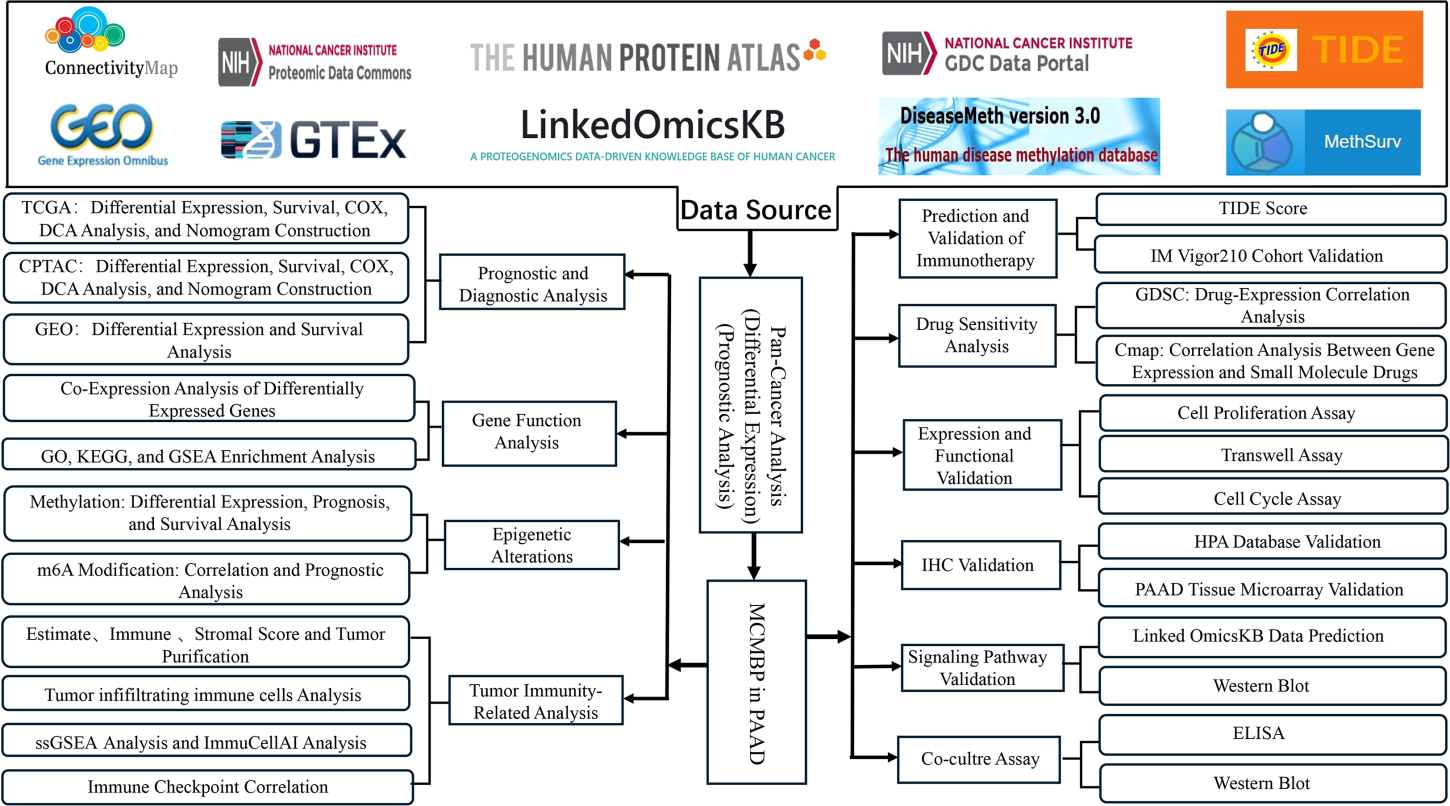
Workflow of the study.

In this study, we employed bioinformatics approaches to investigate the role of MCMBP in PAAD. Leveraging data from TCGA, CPTAC, and other public databases, we evaluated the prognostic significance, epigenetic regulation, immune infiltration patterns, immunotherapy response, and potential therapeutic agents associated with MCMBP. MCMBP expression was validated using western blotting and IHC. Furthermore, by integrating phosphorylation site predictions from the LinkedOmicsKB database with experimental validation, we aimed to elucidate the molecular mechanisms through which MCMBP may influence PAAD progression. The flow chart of our study process is shown in **Figure 1**.

## Materials and methods

### Data acquisition and prognostic model construction

In this study, we obtained expression data and corresponding clinical information for 33 cancer types from The Cancer Genome Atlas (TCGA; https://portal.gdc.cancer.gov/) for pan-cancer analysis. Normal tissue data were sourced from the Genotype-Tissue Expression (GTEx) database (https://gtexportal.org/home/). The RNA-Seq data from TCGA and GTEx (in FPKM format) were unified by converting them to TPM format using a Perl script and subsequently log2(TPM+1) transformed for cross-tissue comparison. For a focused investigation into PAAD, we integrated three independent cohort-s from TCGA, the Clinical Proteomic Tumor Analysis Consortium (CPTAC; https://pdc.cancer.gov/pdc/), and the Gene Expression Omnibus (GEO; https://www.ncbi.nlm.nih.gov/geo/), which included datasets GSE183795, GSE62452, GSE85916, and GSE79668. The microarray data from GEO were preprocessed using the R package “limma” for background correction and quantile normalization. Differential expression analysis was also performed using the “limma” package with thresholds set at false discovery rate (FDR) < 0.05 and |log2 fold change (FC)| > 1. MCMBP expression data and clinical records from TCGA and CPTAC were used to evaluate clinicopathological correlations and prognostic significance. The proteomic data from CPTAC were used directly with their provided normalized abundance values. Kaplan-Meier (KM) survival curves were generated to visualize patient outcomes. The significance of survival differences was assessed using the log-rank test. Univariate and multivariate Cox proportional hazards regression models were employed to identify independent prognostic factors associated with MCMBP expression, with results expressed as hazard ratios (HRs) and 95% confidence intervals (CIs). For Cox regression, MCMBP expression was included as a continuous variable (after log2 transformation). Nomogram models were developed using the “SvyNom” and “rms” packages in R. Model performance was assessed through calibration curves, time-dependent receiver operating characteristic (ROC) analysis and decision curve analysis (DCA). All statistical tests were two-sided, and a P-value < 0.05 was considered statistically significant. All statistical analyses were performed using R version 4.3.3.

### Gene function analysis

Samples were first divided into high- and low-MCMBP expression groups based on the median expression level. Differentially expressed genes (DEGs) were identified using the R package “limma” (version 3.50.3), with an adjusted p-value (FDR) < 0.05 and an absolute log2 fold change > 1 set as the significance thresholds. Functional enrichment analyses—including Gene Ontology (GO), Kyoto Encyclopedia of Genes and Genomes (KEGG), and Gene Set Enrichment Analysis (GSEA)—were carried out with the clusterProfiler R package (v4.6.2). For GO and KEGG enrichment analysis of DEGs, terms with an FDR < 0.05 were considered significantly enriched. For GSEA, the c2.cp.v7.2.symbols.gmt [Curated] gene set was obtained from the MSigDB database (https://www.gsea-msigdb.org). The enrichment results were filtered using the recommended significance thresholds of a nominal p-value < 0.05 and an FDR q-value < 0.25. Furthermore, the STRING database (https://cn.string-db.org) was utilized to identify genes that interact with MCMBP, and the resulting network was then constructed using the top 10 associated genes (13).

### DNA methylation and mRNA modification

DNA methylation levels within the MCMBP promoter were analyzed in both normal and PAAD tissues using the DiseaseMeth (http://diseasemeth.edbc.org/) and TCGA da-tabases. Expression and survival data pertaining to specific DNA methylation sites of MCMBP were retrieved via the gene visualization tool in the MethSurv database (14). For the investigation of MCMBP and m6A modifications, STAR-counts data along with corresponding clinical information for TCGA-PAAD were downloaded. TPM values were log2-transformed after adding 1 to ensure normality. The expression levels of 24 widely recognized m6A regulators (including Writers, Erasers, and Readers) were extracted. The correlation between MCMBP expression and these m6A regulators was evaluated using Pearson correlation analysis. In line with the approach described by Juan Xu et al. (15), who conducted comprehensive molecular characterization and clinical evaluation of m6A regulators across 33 cancer types, we performed statistical analyses using R software (version 4.3.3). All correlation analyses were two-sided, and a threshold of P < 0.05 was applied to determine statisti-cal significance.

### PAAD immune feature analysis and treatment response prediction

To investigate the relationship between MCMBP and the TME in PAAD, we applied the ESTIMATE algorithm to compute ImmuneScore, StromalScore, ESTIMATEScore, and tumor purity (16). We selected 25 immune cell types and performed single sample gene set enrichment analysis (ssGSEA) to calculate enrichment scores per sample. The “GSVA” R package (version 1.46.0) was used for ssGSEA implementation. Samples were ranked by MCMBP expression levels, and heatmaps were generated based on ssGSEA scores. The QUANTISEQ algorithm was used to estimate the infiltration- levels of ten types of tumor-infiltrating immune cells (TIICs) in each sample (17), and statistical comparisons were made between MCMBP expression subgroups. The Wilcoxon rank-sum test was applied to compare immune cell infiltration and immune checkpoint expression between MCMBP-high and MCMBP-low groups, with p < 0.05 considered statistically significant. Based on studies by Auslander et al. (18, 19), we compiled a list of 26 therapeutically relevant immune checkpoints (ICPs) and evaluate-d their correlation with MCMBP expression. Pearson correlation analysis was employed, and the correlation coefficients (R) along with p-values were reported. To predict the clinical response to immunotherapy, the TIDE web tool (http://tide.dfci.harvard.edu/login/) was utilized to analyze TCGA-PAAD expression profiles and compute TIDE scores related to MCMBP (20). These predictions were further validated using the I-Mvigor210 cohort (a cohort of patients with urothelial cancer treated with anti-PD-L1 therapy) (21).

### Drug sensitivity analysis

To identify potential therapeutic agents targeting MCMBP, we integrated drug sensiti-vity data from the GDSC database (https://www.cancerrxgene.org/) with MCMBP expression profiles from PAAD patients in TCGA. Using the oncoPredict R package (version 0.2), drug sensitivity scores were computed and their correlation with MCMBP expression levels was assessed using Spearman’s rank correlation method. Drugs showing a significant correlation (Spearman’s p < 0.05) were ranked by the magnitude of their correlation coefficients and visualized using R version 4.3.3. Additionally, we employed the Connectivity Map (CMap) database (https://clue.io/) to explore connections among small molecules, gene expression, and disease phenotypes. Based on clinical treatment outcomes, PAAD patient samples were categorized into progressive/stable disease (PD/SD) and partial/complete response (PR/CR) groups. DEGs between these groups were identified using the ‘limma’ R package, with p < 0.05 and |log2 fold change| > 1 set as the significance thresholds, and the resulting DEG set was correlate-d with MCMBP (p < 0.05). Using these gene sets and the CMap platform, we screened for small molecule drugs potentially targeting MCMBP in PAAD. Compounds with a negative connectivity score (norm_cs < 0) in CMap were considered potential MCMBP inhibitors.

### Cell culture

Human pancreatic ductal epithelial cells (HPNE) and PAAD cell lines (MIA PaCa-2, BxPC-3, PANC-1, Capan-2, AsPC-1, Jurkat) were purchased from Wuhan ProCell Biotechnology Co., Ltd. Cells were cultured at 37 °C in a humidified atmosphere of 95% air and 5% CO_2_, using modified Dulbecco’s Modified Eagle Medium (DMEM), RPMI-1640, and McCoy’s 5A medium (all from Procell, CN), supplemented with 10% fetal bovine serum (FBS, Procell, CN), 2.5% horse serum (HS, Procell, CN), and 100 U/mL penicillin and streptomycin (Procell, CN).

### Western blotting analysis

Total cellular proteins were extracted using RIPA lysis buffer (Beyotime Biotechnolog-y, Shanghai, China) supplemented with protease inhibitors. Equal amounts of protein samples (30µg) were separated on a 12% SDS-PAGE gel and subjected to sodium dodecyl sulfate-polyacrylamide gel electrophoresis (SDS-PAGE) under denaturing conditions (20–50µg). The proteins were then transferred onto PVDF membranes. Primary antibodies were applied following the manufacturer’s instructions (**Supplementary Table S1**). Finally, GAPDH was used as a loading control, and protein band intensities were quantified and normalized using ImageJ software (version 1.48). The Western blot image shown is representative of multiple independent experiments.

### Lentiviral infection

The MCMBP overexpression lentivirus was purchased from GeneChem (Beijing, Chi-na), and the shRNA primers were designed on the Merck website (http://www.sigmaaldrich.com). The pLKO.1TRC vector was digested with restriction enzymes, and the digestion products were ligated with the amplified fragments. Following ligation, colony transformation, screening, sequencing, and recombinant plasmid extraction were performed. PEI was used for transfection, mixing PS, PM, and plasmid DNA in serum-free DMEM. The mixture was transfected into 293T cells for 24 hours. Lentivirus at a concentration of 1×10^7^ transducing units (TU) was used to infect 1×10^5^ target cells, with an empty vector lentivirus as the negative control. After transfection, the cells underwent 5 weeks of antibiotic selection, after which they were collected for further analysis.

### Cell cycle analysis

Following transfection, target cells were selected using penicillin-streptomycin solution for 48 hours. The cells were fixed with 75% ethanol and stained with propidium iodide solution containing RNaseA at room temperature for 30 minutes. Cell cycle analysis was performed using a cell cycle analysis kit (Beyotime, Jiangsu, China). According to the kit instructions, 1×10^6^ cells were stained and analyzed with a flow cytometer (Agilent NovoCyte3110, California, USA). Data was processed using the NovoExpress software version 1.5.0 (Agilent, California, USA). The results are presented as the mean ± SD from three independent experiments.

### Cell formation and Transwell assays

After transfection, cells were seeded into 6-well plates at a density of 3×10³ cells per well and incubated in a humidified 5% CO_2_ incubator at 37 °C for 2 weeks. The medium was replaced every 3 days, and cell conditions were regularly observed. In the Transwell assay, 8×10^5^ cells per well and 100 μL serum-free medium were added to the upper chamber, while 600 μL 20% FBS medium was placed in the lower chamber. The plate was incubated at 37 °C for 24 hours. For the Matrigel Transwell assay, a layer of Matrigel matrix (Corning) was first applied to the upper chamber (diluted 8:1 with serum-free medium), and 100 μL serum-free medium was added to both the upper and lower chambers. The system was incubated at 37 °C for 36 hours. After incubation, the medium and floating cells were removed, and the cells were washed twice with PBS, fixed with 4% paraformaldehyde, and stained with 0.5% crystal violet solution for 15 minutes. After staining, images were captured, and the number of cells was counted. The results are presented as the mean ± SD from three independent experiments.

### Tissue microarray and IHC

A PAAD tissue microarray (HPanA120Su02) was purchased from Shanghai Outdo Biotech Co., Ltd. (Shanghai, China). MCMBP (dilution 1:200) was used as the primary antibody. This study was approved by the Ethics Committee of Shanghai Outdo Biotech (Approval No. XSW-02-02). A total of 70 PAAD tissue samples were collected from the tissue microarray, including 47 paired normal tissues, to assess MCMBP expression levels. Immunohistochemistry (IHC) scoring was based on staining intensity (no staining: 0, weak staining: 1, moderate staining: 2, strong staining: 3) and the percentage of positive cells (<25%: 0, 25-50%: 1, 50-75%: 3, >75%: 4), with a total score range of 0 to 12. The expression levels of MCMBP were compared using a t-test. All analyses were based on these independent tissue samples.

### Cell co-culture and ELISA assays

Control, MCMBP-overexpressing, and MCMBP-knockdownAsPC-1 and PANC-1 cells were seeded in 24-well plates at a density of 1 × 10^5^ cells per well. Cells were cultured for 24 hours in their respective complete media RPMI-1640 for AsPC-1 and DMEM for PANC-1, both supplemented with 10% FBS and 1% P/S. Jurkat T cells were then added directly to the tumor cells at a density of 4 × 10^5^ cells per well. After a 2 hour stabilization period, T-cell activation was induced by adding soluble anti-CD3 antibody (2μg/mL, Elabscience, Wuhan, CN), soluble anti-CD28 antibody (1μg/mL, Elabscience, Wuhan, CN), and Goat Anti-Mouse IgG (5μg/mL, Elabscience, Wuhan, CN) to the culture medium. Following 24 hours of activation, the cell culture supernatant was collected, and the concentration of secreted IFN-γ was quantified using a Human IFN-γ ELISA Kit (Elabscience, Wuhan, CN) according to the manufacturer’s instructions. Concurrently, Jurkat cells were harvested for subsequent Western blotting analysis. Data are presented as the mean ± SEM from three independent experiments. Statist-ical significance was determined using two-way ANOVA with multiple comparisons test.

### Statistical analysis

Statistical analyses were conducted using R (version 4.3.3) and GraphPad Prism 9.0 (GraphPad Software, La Jolla, CA, USA). For *in vitro* experiments, continuous data are expressed as mean ± standard deviation (SD). Comparisons between two groups of normally distributed data were performed using unpaired or paired two-tailed Student’s t-tests, as appropriate. Comparisons among multiple groups were analyzed by one-way or two-way ANOVA, followed by Tukey’s *post-hoc* test for multiple comparisons. A P-value < 0.05 was considered statistically significant (*P < 0.05; **P < 0.01; ***P < 0.001). All statistical tests were two-sided.

## Results

### Pan-cancer analysis of MCMBP and its overexpression predicting poor prognosis in PAAD

**Figure 2:**
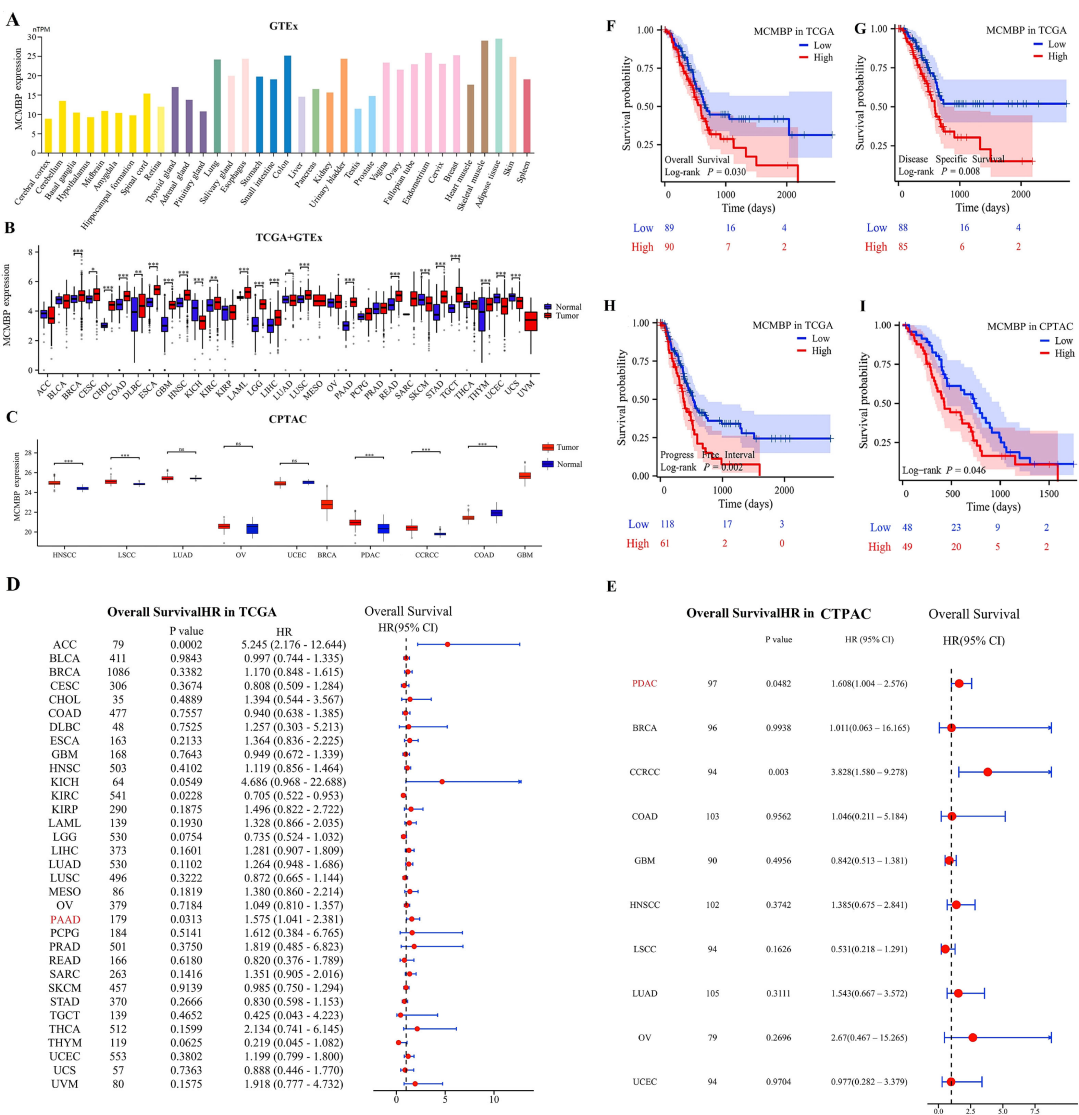
Expression levels and prognostic significance of MCMBP. **(A–C)** Expression of MCMBP in tumor tissues versus normal tissues based on HPA, TCGA+GTEx, and CPTAC datasets; **(D, E)** Univariate Cox regression analysis of MCMBP expression across various cancer types using TCGA and CPTAC datasets; **(F–I)** KM survival analysis of MCMBP expression based on TCGA and CPTAC datasets. *P<0.05, **P<0.01, ***P<0.001, ns not significant (indicating no statistical significance).

Analysis of the HPA dataset indicated that MCMBP expression varies across normal tissues, with particularly high levels detected in skeletal muscle, adipose tissue, endometrium, colon, and breast tissue (**Figure 2A**). Pan-cancer analysis using data from TCGA and GTEx revealed widespread dysregulation of MCMBP expression in multiple cancer types. Specifically, MCMBP was significantly upregulated in BRCA, CHOL, COAD, ESCA, GBM, HNSC, LAML, LGG, LIHC, LUSC, PAAD, READ, STAD, TGCT, and THYM compared to normal tissues (P<0.05). In contrast, significant downregulation was observed in KICH, SKCM, UCEC, and UCS (P<0.05, **Figure 2B**). These findings were further corroborated by the CPTAC database, which also showed markedly elevated MCMBP expression in HNSC, LSCC, PDAC, and CCRCC (**Figure 2C**). Collectively, these results suggest that aberrant MCMBP expression is closely associated with cancer progression.

To explore the prognostic significance of MCMBP expression, we performed univariate Cox regression analysis across 33 cancer types to assess its correlation with overall survival (OS). In the TCGA cohort, MCMBP overexpression was found to be significantly associated with poorer OS in ACC (P<0.001) and PAAD (P=0.0313, **Figure 2D**). Similarly, data from the CPTAC cohort further corroborated this finding, demonstrating that elevated MCMBP expression was significantly associated with worse OS in PDAC (P=0.0482) and CCRCC (P=0.003, **Figure 2E**). Taken together, these findings indicate that high MCMBP expression is correlated with poor prognosis in PAAD and PDAC. To further evaluate the prognostic value of MCMBP in PAAD, we stratified patients into high-expression and low-expression subgroups and conducted KM survival analyses. In the TCGA cohort, MCMBP overexpression was significantly associated with shorter OS, disease-specific survival (DSS), and progression-free interval (PFI) (**Figures 2F–H**). Consistently, in the CPTAC cohort, the high-expression subgroup also exhibited significantly poorer OS (**Figure 2I**).

To validate the association between MCMBP and unfavorable prognosis in PAAD, we analyzed PAAD patient samples from the GEO database. MCMBP expression was elevated in tumor tissues compared to normal controls in both the GSE62452 (**Supplementary Figure S1A**) and GSE183795 (**Supplementary Figure S1B**) cohorts. Additionally, in the GSE79668 (**Supplementary Figure S1C**) and GSE85916 (**Supplementary Figure S1D**) cohorts, high MCMBP expression was significantly correlated with worse OS. These multi-database, multi-cohort analyses further reinforce the potential of MCMBP as a prognostic biomarker for unfavorable outcomes in PAAD.

### Clinical pathological analysis and development of the prognostic model

**Figure 3:**
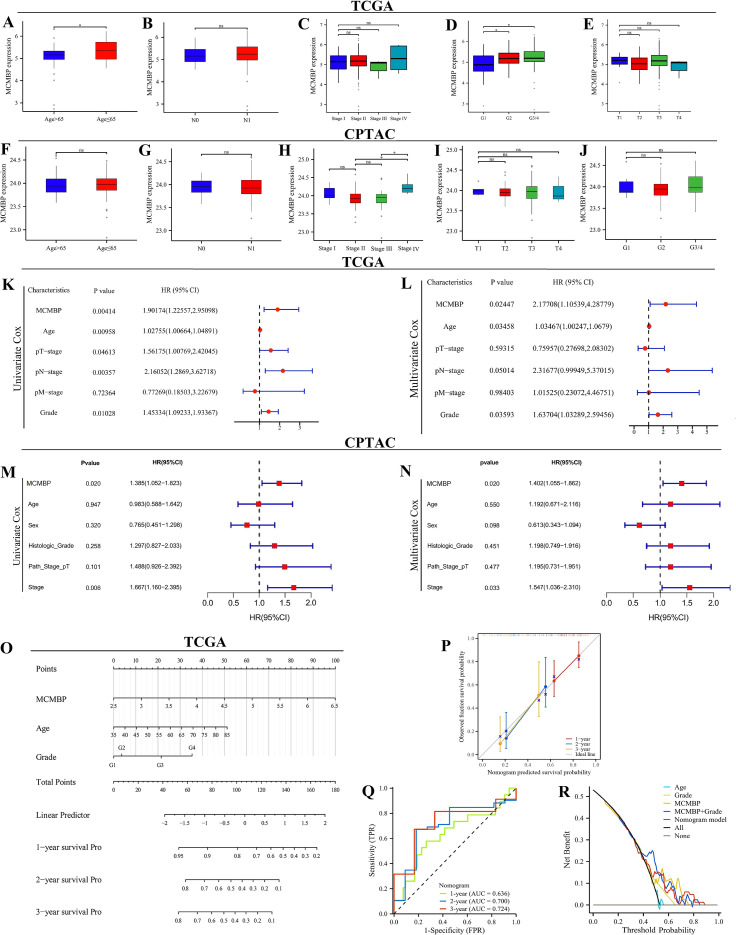
Clinical pathological feature analysis and prognostic model construction. **(A–J)** Correlation of MCMBP with clinical pathological features in the TCGA and CPTAC datasets; **(K, L)** Univariate and multivariate Cox regression analysis in the TCGA cohort; **(M, N)** Univariate and multivariate Cox regression analysis in the CPTAC cohort; **(O)** Nomogram models in the TCGA cohort; **(P)** Calibration curves in the TCGA cohort; **(Q)** ROC curves in the TCGA cohort; **(R)** DCA curves in the TCGA cohort. *P < 0.05, ns not significant (indicating no statistical significance).

In the TCGA cohort, MCMBP expression increased significantly with higher tumor grade (**Figures 3A–E**). Similarly, in the CPTAC cohort (**Figures 3F–J**), MCMBP expression levels were significantly elevated with advancing tumor stage. Univariate Cox regression analysis revealed that, in the TCGA cohort (**Figure 3K**), MCMBP expression, T stage, N stage, and tumor grade were all significantly associated with PAAD prognosis. In the CPTAC cohort (**Figure 3M**), MCMBP expression and tumor stage were significantly correlated with prognosis. Multivariate analysis further confirmed that MCMBP expression and tumor grade were independent prognostic factors in the TCGA cohort (**Figure 3L**). Similarly, MCMBP expression and tumor stage were independent predictors in the CPTAC cohort (**Figure 3N**). These results underscore the independent prognostic value of MCMBP in PAAD. To facilitate clinical translation, we integrated the independent prognostic factors—age, tumor grade, tumor stage, and MCMBP expression—into a nomogram for predicting OS in both TCGA and CPTAC cohorts (**Figure 3O**; **Supplementary Figure S2A**). Calibration curves demonstrated high predictive accuracy for 1-, 2-, and 3-year OS in the TCGA training cohort (**Figure 3P**). In the CPTAC validation cohort, the model showed moderate accuracy (**Supplementary Figures S2B, C**). By comparison, the nomogram achieved higher predictive performance in the TCGA cohort, with AUCs of 0.700 and 0.724 for 2- and 3-year OS (**Figure 3Q**). Decision curve analysis further indicated that MCMBP expression provided substantial clinical net benefit in the TCGA cohort (**Figure 3R**) and remained informative in the CPTAC cohort (**Supplementary Figure S2D**). In summary, these findings support MCMBP as a prognostic biomarker in PAAD and propose a clinically applicable nomogram for risk stratification. The observed variations in model performance between cohorts highlight the need for further validation and refinement.

### Biological function analysis of MCMBP

**Figure 4:**
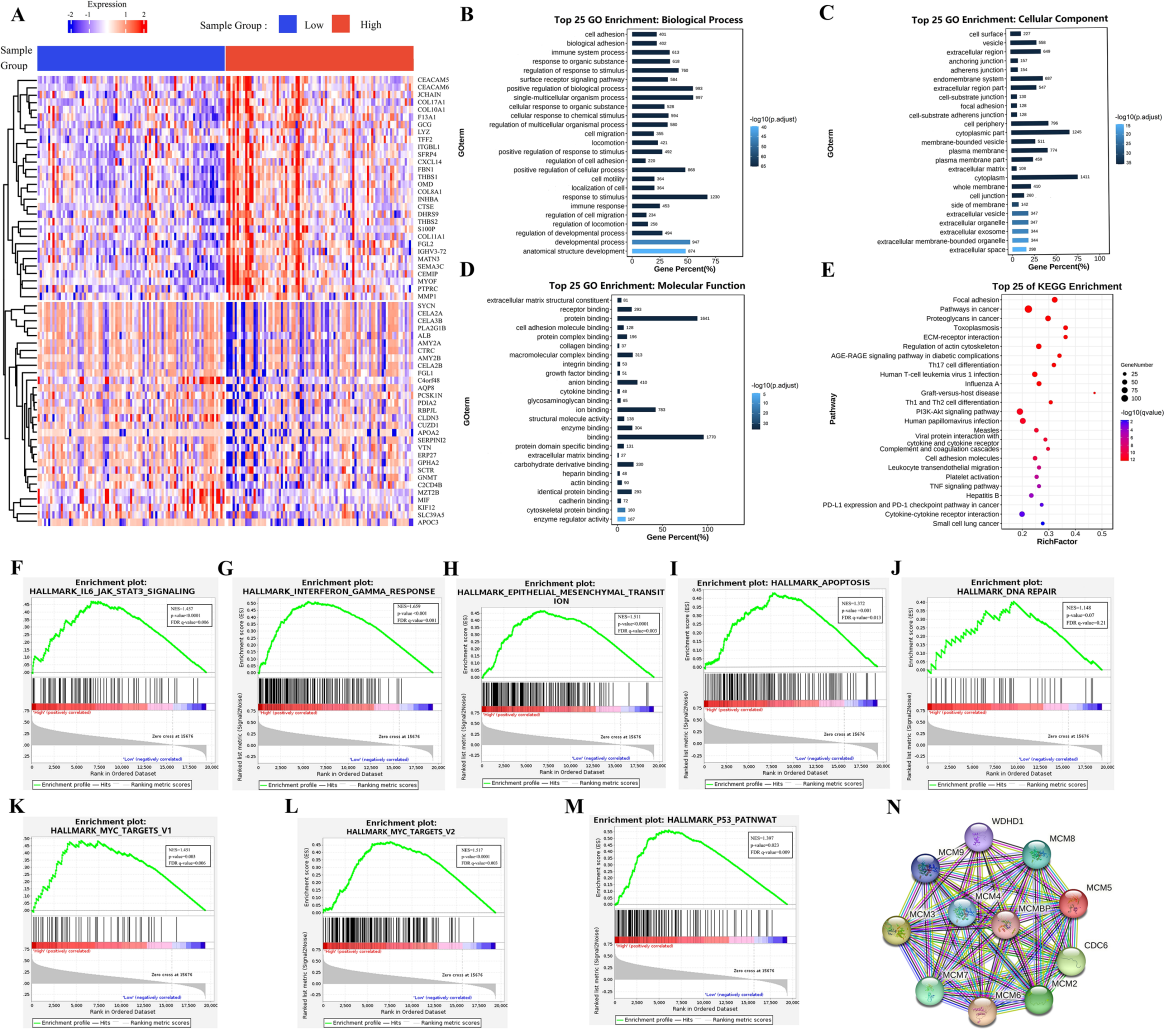
Biological function analysis of MCMBP. **(A)** The expression of the top 100 most significantly up and down-regulated genes analysis between high and low MCMBP expression groups using the limma package; **(B)** Biological processes associated with MCMBP gene functions, **(C)** Cellular components of MCMBP gene functions, **(D)** Molecular functions associated with MCMBP gene functions. **(E)** KEGG enrichment analysis between groups expressing high and low levels of MCMBP. **(F–M)** GSEA analysis of the group expressing high levels of MCMBP including immune response, cell migration, cell cycle, and chromosomal instability signals. **(N)** PPI analysis of MCMBP-associated proteins.

Limma analysis identified 5,640 upregulated and 116 downregulated genes associated with MCMBP expression. The heatmap (**Figure 4A**) displays the expression of the top 30 most significantly up and down-regulated genes. GO enrichment analysis (**Figures 4B–D**) showed that MCMBP may enhance tumor-stroma interactions through the regulation of extracellular matrix structural constituents and focal adhesion pathways. Furthermore, MCMBP appears to promote cell proliferation and survival via receptor binding, protein binding, and growth factor binding signaling pathways, suggesting a potential role in facilitating tumor cell migration and invasion. Enrichment of specific protein domain binding also implies a role for MCMBP-mediated epigenetic regulation in tumor progression. KEGG pathway analysis (**Figure 4E**) revealed that MCMBP regulates the cell cycle through activation of the PI3K-Akt signaling pathway, supporting its function in promoting proliferation. Additionally, MCMBP may modulate immune responses by influencing Th17 cell differentiation, Th1/Th2 cell balance, T-cell receptor signaling, and the PD-L1/PD-1 checkpoint pathway, potentially contributing to immune evasion. These findings were corroborated by GSEA, which showed significant enrichment of MCMBP-upregulated genes in pathways associated with tumor immune escape, cell migration and invasion, cell cycle progression, chromosomal instability, and transcriptional regulation (**Figures 4F–M**). Protein-protein interaction (PPI) network analysis identified interactions between MCMBP and MCM2–9 as well as WDHD1 (**Figure 4N**). Together, these results suggest that MCMBP plays a multifaceted and critical role in promoting PAAD progression.

### Epigenetics and prognostic analysis

**Figure 5:**
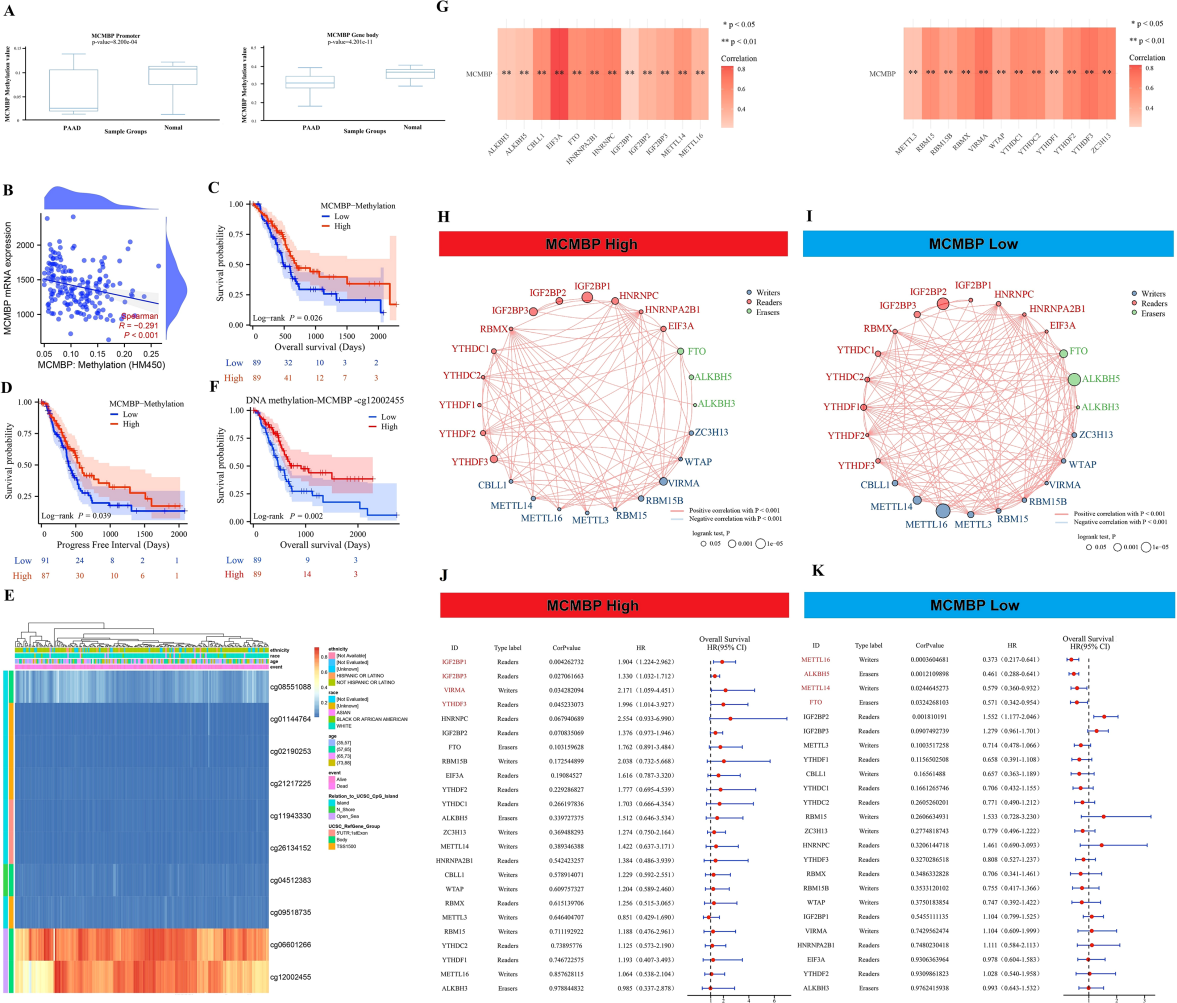
Epigenetics and prognostic analysis. **(A)** Methylation levels of the MCMBP promoter and gene body in tumor and adjacent normal tissues; **(B)** Correlation analysis between MCMBP methylation status and its expression levels; **(C, D)** OS and PFI analysis based on MCMBP methylation levels; **(E)** Expression of MCMBP at different methylation sites; **(F)** OS analysis of the MCMBP-cg12002455 methylation site; **(G)** Correlation analysis between MCMBP and m6A modification-related genes; **(H, I)** Prognostic correlation analysis of MCMBP and m6A modification-related genes in the high-expression group; **(J, K)** Prognostic correlation analysis of MCMBP and m6A modification-related genes in the low-expression group. *P < 0.05, **P < 0.01.

Based on analyses using the DiseaseMeth and TCGA databases, we found that MCMBP methylation levels are significantly higher in normal tissues than in tumor tissues (**Figure 5A**). Further correlation analysis demonstrated a significant negative association between the methylation status of MCMBP and its expression levels (**Figure 5B**). To evaluate the prognostic value of MCMBP methylation in PAAD, KM survival analysis was performed. The results for OS and PFI revealed that low methylation levels of MCMBP were associated with poor prognosis (**Figures 5C, D**), suggesting that the methylation status of MCMBP may serve as a potential prognostic biomarker for PAAD. To further investigate the methylation characteristics of MCMBP, we performed a comprehensive analysis of multiple CpG methylation sites using the MethSurv database (**Figure 5E**). Heatmap results indicated that sites cg06601266 and cg12002455 exhibited high methylation levels, while sites cg01144764, cg02190253, cg2127225, cg11943330, and cg26134152 displayed low methylation levels. Further prognostic analysis demonstrated that the expression of MCMBP at the cg12002455 site was significantly associated with patient outcomes (**Figure 5F**). These findings suggest that higher methylation levels of MCMBP are correlated with better prognosis. In addition, correlation analysis of 24 genes involved in m6A modification revealed that MCMBP was significantly associated with these genes, with statistically significant differences observed (**Figure 5G**). MCMBP expression was categorized into high and low groups, and both Log-rank P tests and univariate Cox regression analyses were performed (**Figures 5H–K**). Results indicated that in the high MCMBP expression group, IGF2BP1, IGF2BP3, VIRMA, and YTHDF3 were significantly upregulated (HR>1). This co-expression pattern suggests that MCMBP-high tumors are associated with a state that favors m6A modification, which is linked to poor patient survival. Conversely, low MCMBP expression was associated with higher levels of m6A “erasers” (ALKBH5, FTO) (HR<1), implying a potential tendency for m6A removal in this context, which correlates with more favorable clinical outcomes. In summary, our integrated analysis indicates that the low DNA methylation of MCMBP and its associated pro-tumorigenic m6A regulator profile may collectively constitute an epigenetic signature linked to adverse prognosis in PAAD.

### The relationship between MCMBP and immunity in PAAD

**Figure 6:**
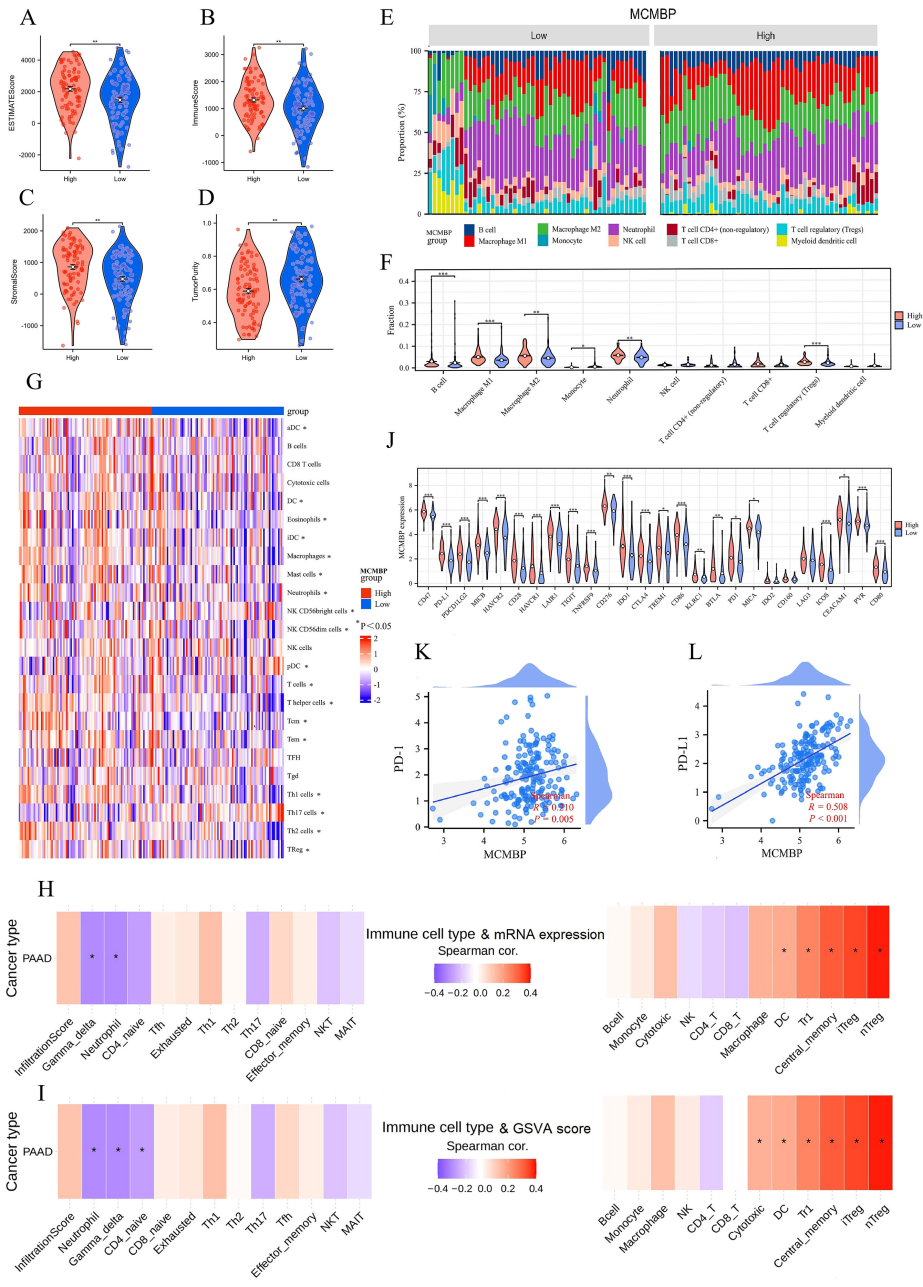
The relationship between MCMBP and immunity in PAAD. **(A–D)** ESTIMATE algorithm analysis of MCMBP immune infiltration; **(E–G)** Differential analysis of TIICs between MCMBP high and low expression groups using QUANTISEQ and ssGSEA; **(H)** Integrated analysis of MCMBP mRNA expression levels with ImmuCellAI analysis; **(I)** GSVA combined with ImmuCellAI analysis; **(J)** Differential expression analysis of ICPs between MCMBP subgroups; **(K, L)** Correlation analysis between MCMBP expression and PD-1/PD-L1 levels. *P<0.05, **P<0.01, ***P<0.001.

Previous KEGG/GO analyses indicated a potential role for MCMBP in tumor immunity. ESTIMATE analysis showed that high MCMBP expression was associated with higher ESTIMATE, Immune, and Stromal scores, and lower Tumor Purity (**Figures 6A–D**), suggesting its potential involvement in modulating the TME. To further explore the link between MCMBP and immune infiltration, we performed QUANTISEQ and ssGSEA analyses. QUANTISEQ revealed significantly elevated infiltration of M2 macrophages, Tregs, and neutrophils in the high MCMBP expression group (**Figures 6E, F**, all p<0.05). ssGSEA indicated positive correlations between MCMBP expression and infiltration of B cells, T helper cells, Tregs, Th2 cells, and T cells, and negative correlations with macrophages, mast cells, neutrophils, and dendritic cells (**Figure 6G**, all p<0.05). These findings suggest that MCMBP expression in PAAD might actively participate in immunosuppressive cell infiltration. Further analysis using ImmuCellAI combined mRNA expression showed significant positive correlations between MCMBP expression and immunosuppressive Treg subsets, including iTreg, nTreg, and Tr1 cells (**Figure 6H**; **Supplementary Figure S3B**, all p<0.05), suggesting that MCMBP may enhance Treg recruitment and function. GSVA integrated with ImmuCellAI also revealed positive associations between MCMBP activity and iTreg, nTreg, Tr1, and dendritic cells, and negative correlations with gamma delta T cells and neutrophils (**Figure 6I**; **Supplementary Figure S3A**, all p<0.05). These results imply that MCMBP may facilitate immunosuppression through IL-6 signaling, FoxP3 expression, PD-1/PD-L1 checkpoint activation, and JAK-STAT or NF-κB pathways. Among 26 immune checkpoints (ICPs) analyzed, most—including PD-1 and PD-L1—were upregulated in the high MCMBP subgroup (**Figure 6J**). Correlation analysis confirmed positive associations between MCMBP expression and both PD-1 and PD-L1 (**Figures 6K, L**). In summary, MCMBP may contribute to immune evasion in PAAD by enhancing immunosuppressive cell infiltration and upregulating key immune checkpoints.

### Immunotherapy prediction and drug sensitivity analysis

**Figure 7:**
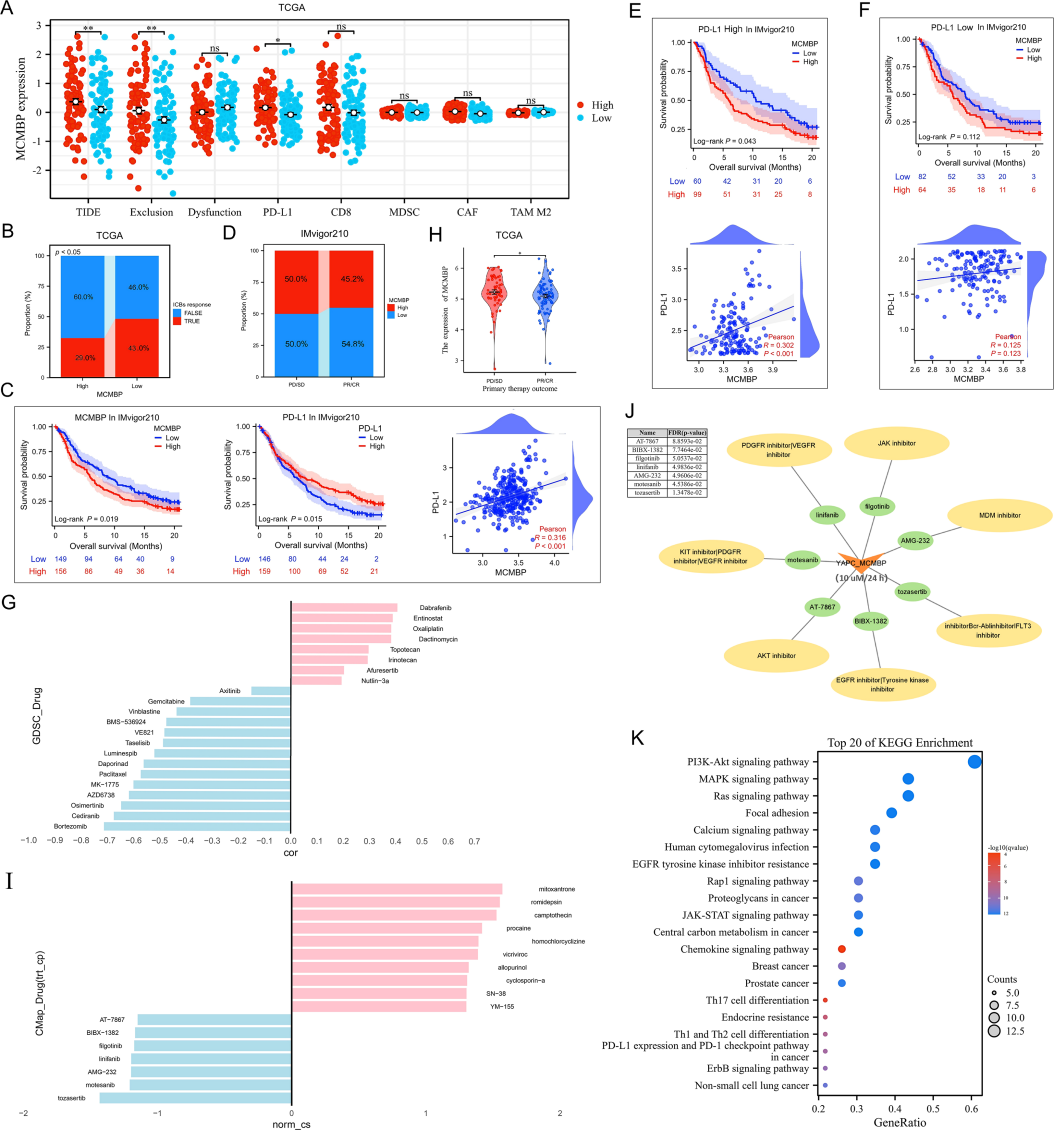
Immunotherapy prediction and drug sensitivity analysis **(A, B)** TIDE, Exclusion, PD-L1 scores, and ICB response rates in low and high MCMBP subgroups in TCGA; **(C)** OS and correlation analysis of MCMBP and PD-L1 in the IMvigor210 cohort; **(D)** Proportion of high and low MCMBP expression correlated with clinical immunotherapeutic response subgroups in the IMvigor210 cohort; **(E, F)** OS and correlation analysis of MCMBP in high and low PD-L1 expression groups; **(G)** Drug correlation analysis in GDSC; **(H)** Primary therapy outcome of MCMBP in TCGA-PAAD; **(I)** CMap drug correlation analysis; **(J)** Small molecule drugs associated analysis; **(K)** KEGG analysis was performed on the gene set targeted by the CMap-identified drugs. *P < 0.05, **P < 0.01, ns not significant (indicating no statistical significance).

The efficacy of anti-PD-L1 immunotherapy in PAAD is influenced by factors such as tumor mutation burden (TMB), PD-L1 expression, and Treg expression levels (21–24). Based on our previous results (**Figures 6J–L**), we employed the TIDE algorithm to evaluate the effect of MCMBP expression on response to immune checkpoint blockade (ICB), including anti-PD-1 and anti-PD-L1 therapies. TIDE, Exclusion, and PD-L1 scores were significantly higher in the high MCMBP expression group (**Figure 7A**, all P < 0.05). Further analysis of ICB response rates revealed that among patients with high MCMBP expression, were predicted to respond to treatment while 60% were predicted non-responders. In contrast, the low MCMBP expression group showed a significantly higher predicted response rate of 43%, with non-responders reduced to 46% (**Figure 7B**, P < 0.05). These results suggest that high MCMBP expression correlates with elevated TIDE and Exclusion scores and reduced ICB efficacy, whereas low MCMBP expression is associated with lower PD-L1 scores and improved ICB response.

To validate these findings, we analyzed the IMvigor210 cohort dataset. Both MCMBP and PD-L1 expression significantly associated with OS, with a positive correlation between them (**Figure 7C**). A greater proportion of patients with low MCMBP expression were found in the partial response (PR) and complete response (CR) groups, further supporting that low MCMBP may predict better immunotherapy sensitivity (**Figure 7D**). Upon stratification by PD-L1 expression, Kaplan–Meier survival and correlation analyses showed more pronounced survival differences and stronger correlations in the high-PD-L1 group than in the low-PD-L1 group, implying a potential synergy between MCMBP expression and anti-PD-L1 treatment (**Figures 7E, F**).

Analysis of the GDSC database indicated that MCMBP expression was negatively correlated with drug IC50 for Gemcitabine, Paclitaxel, Bortezomib, and Cediranib (**Figure 7G**), suggesting that high MCMBP expression may increase responsiveness to gemcitabine–paclitaxel combination chemotherapy. To identify potential MCMBP-targeting compounds, we analyzed differentially expressed genes in PR/CR groups from TCGA-PAAD (**Figure 7H**; **Supplementary Figures S4A, B**) and queried the Connectivity Map (CMap) database. Compounds with negative connectivity scores (norm_cs < 0) were considered potential MCMBP inhibitors, including Tozasertib, Motesanib, AMG-232, Linifanib, Filgotinib, BIBX-1382, and AT-7867 (**Figure 7I**). Using YAPC cells with high MCMBP expression treated at 10 µM for 24 hours, we further screened for sensitive agents and found that Tozasertib, Motesanib, AMG-232, Linifanib, and Filgotinib showed therapeutic potential (FDR/p-value ≤ 0.05, **Figure 7J**). To further validate the potential functions of these candidate compounds, KEGG analysis was performed on the gene set targeted by the CMap-identified drugs. The results revealed significant enrichment of multiple cancer-related signaling pathways, including the PI3K-Akt, MAPK, Ras, and JAK-STAT pathways, as well as the PD-L1 expression and PD-1 checkpoint pathway in cancer (**Figure 7K**). The concordance between the pathways targeted by these effective MCMBP-inhibiting compounds and previously identified MCMBP-associated functions collectively (**Figure 4E**) suggests that the tumor-promoting role of MCMBP in PAAD is likely mediated through the regulation of these specific signaling pathways.

### Knockdown of MCMBP affects the proliferation, migration, and invasion of PAAD cells

**Figure 8:**
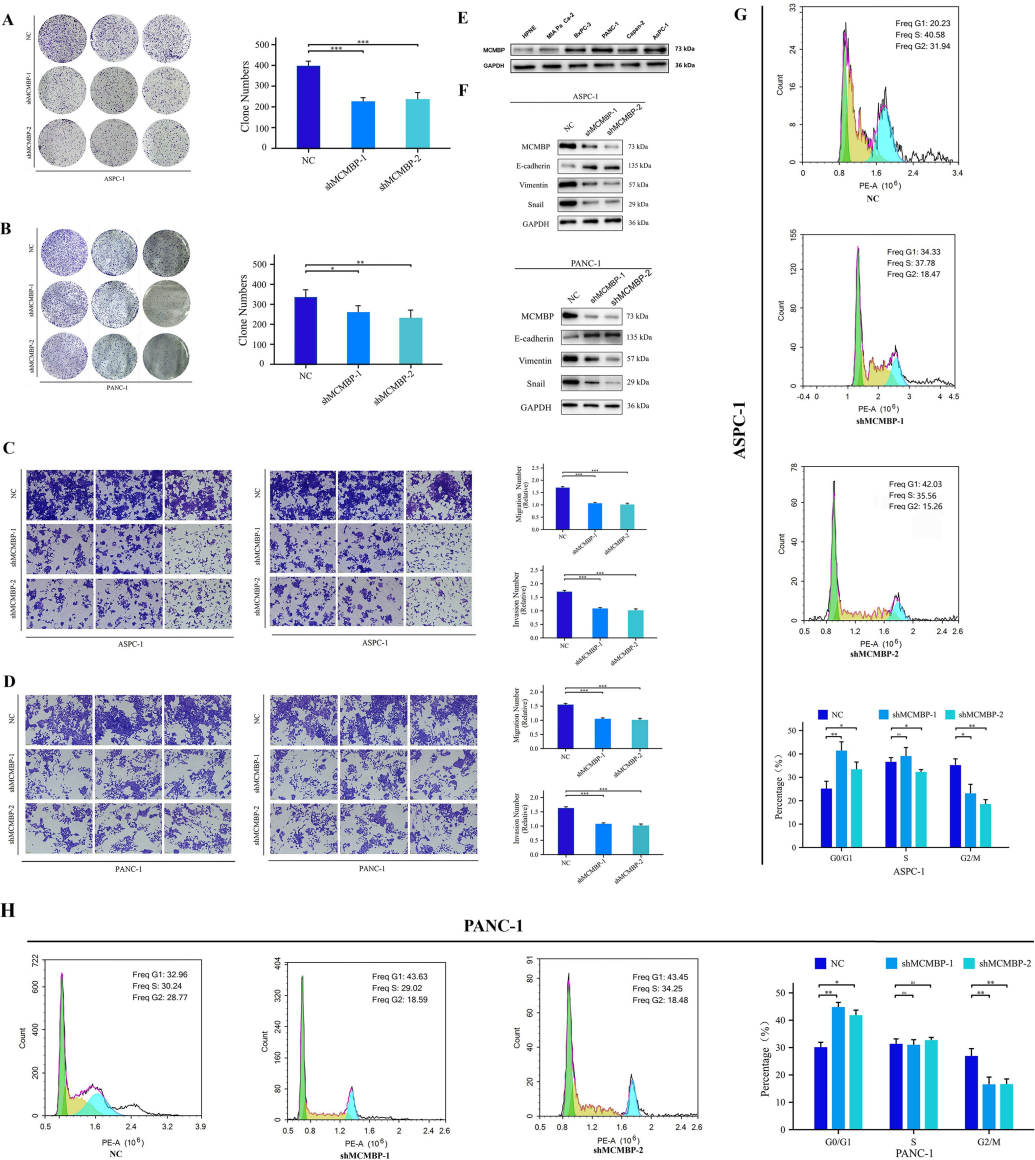
Knockdown of MCMBP affects the proliferation, migration, and invasion of PAAD cells. **(A, B)** Colony formation assay in ASPC-1 and PANC-1 cells; **(C, D)** Representative images and statistical analysis of migration and invasion assays in ASPC-1 and PANC-1 cells; **(E)** Expression levels of MCMBP across cell lines HPNE, MIA PaCa-2, BxPC-3, PANC-1, Capan-2, and ASPC-1; **(F)** Expression levels of EMT-related proteins in ASPC-1 and PANC-1 cell lines; **(G, H)** Flow cytometry analysis in ASPC-1 and PANC-1 cells. *P < 0.05, **P < 0.01, ***P < 0.001, ns not significant (indicating no statistical significance).

To examine MCMBP expression in PAAD, we performed Western blot analysis on five PAAD cell lines and a normal pancreatic ductal epithelial cell line (HPNE). MCMBP protein levels were low in HPNE cells but significantly elevated in ASPC-1 and PANC-1 cells (**Figure 8E**). To explore the functional role of MCMBP, we knocked down its expression in ASPC-1 and PANC-1 cells using a lentiviral-based approach. Colony formation assays revealed that MCMBP knockdown significantly suppressed proliferation in both cell lines (**Figures 8A, B**), suggesting a potential role for MCMBP in promoting tumor cell growth. Flow-cytometric cell-cycle analysis showed that MCMBP depletion impeded the G1–S phase transition and reduced the proportion of cells in G2/M phase (**Figures 8G, H**), suggesting that MCMBP may regulate cell cycle progression. Furthermore, migration and invasion capabilities were significantly impaired upon MCMBP knockdown (**Figures 8C, D**). Western blot analysis of epithelial-mesenchymal transition (EMT) markers indicated that MCMBP silencing increased the expression of E-cadherin and decreased levels of Vimentin and Snail (**Figure 8F**). These results suggest that MCMBP may facilitate PAAD cell proliferation by promoting nuclear transport of the MCM complex and enabling S-phase entry, while also enhancing migratory and invasive capacities through activation of the EMT process.

### IHC analysis of MCMBP expression in pancreatic adenocarcinoma

**Figure 9:**
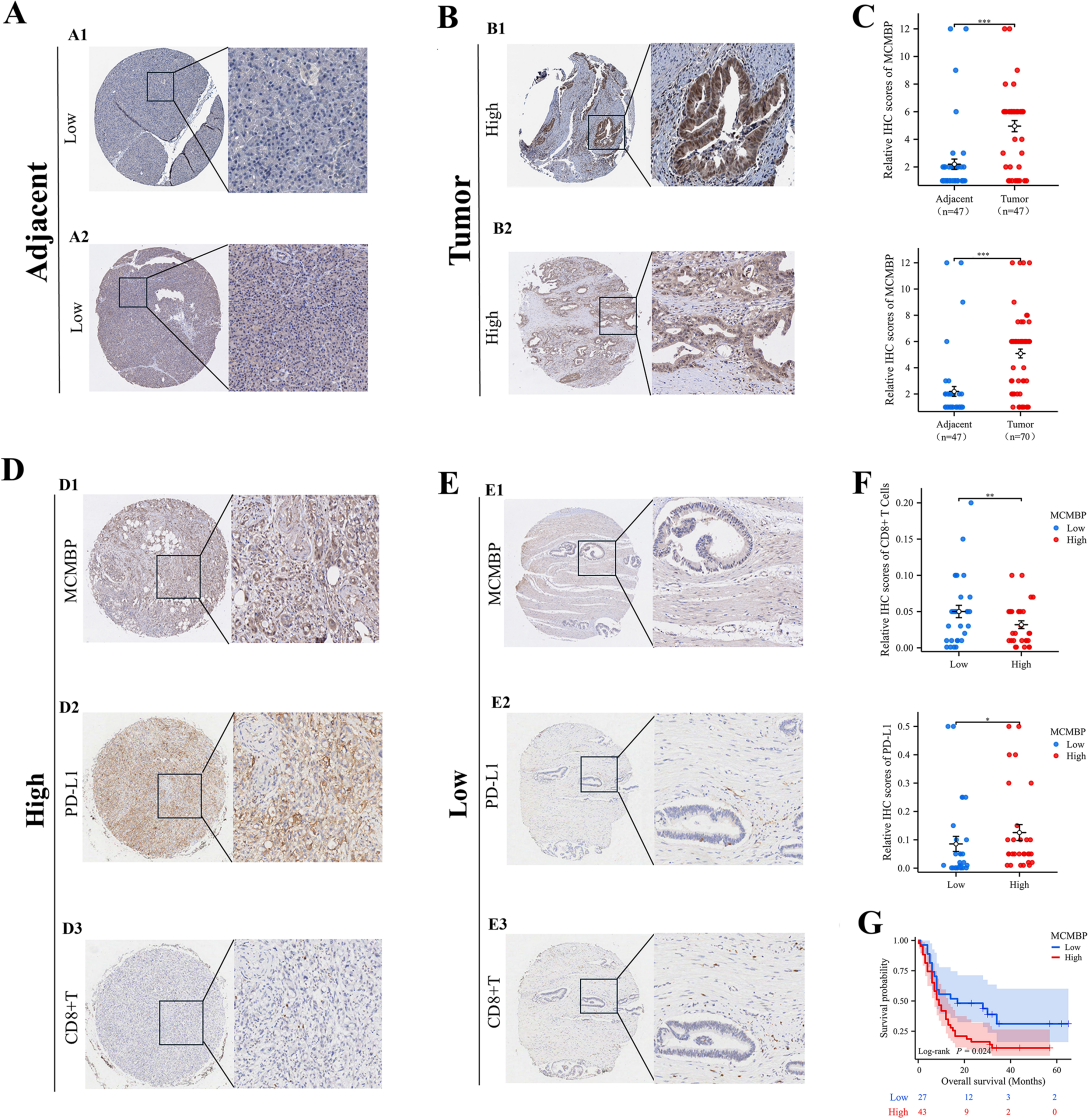
IHC analysis of MCMBP expression in PAAD. **(A, B)** Representative IHC images of MCMBP expression in PAAD and normal tissues; **(C)** Statistical analysis of relative IHC scores for MCMBP; **(D, E)** IHC analysis of MCMBP, PD-L1, and CD8+ T cell expression levels in PAAD; **(F)** Statistical analysis of relative IHC scores for CD8+ T cells and PD-L1; **(G)** OS of PAAD patients stratified by MCMBP expression levels. *P < 0.05, **P < 0.01, ***P < 0.001.

To investigate MCMBP expression in PAAD, we performed immunohistochemical (IHC) staining and scoring on tissue samples from 117 PAAD patients, including 47 paired tumor and adjacent non-tumor samples and 23 unpaired tumor samples (**Figures 9A2, B2**). We also analyzed MCMBP expression in PAAD and normal tissues from the Human Protein Atlas (HPA) database (**Figures 9A1, B1**). The results showed that MCMBP expression was significantly higher in PAAD tissues compared to normal tissues (**Figure 9C**). Further survival analysis integrating IHC scores with clinical data revealed that higher MCMBP expression was associated with shorter patient survival (**Figure 9G**). To assess whether MCMBP may contribute to immune escape in PAAD, we compared PD-L1 and CD8+ T-cell infiltration based on IHC scores. Tumors with high MCMBP expression exhibited significantly higher PD-L1 expression (**Figure 9D2**) and lower CD8+ T-cell infiltration (**Figure 9D3**) compared to those with low MCMBP expression (**Figure 9E**), and all differences were statistically significant (**Figure 9F**). These findings suggest that aberrant overexpression of MCMBP in PAAD may be associated with poor prognosis and potential involvement in immune escape mechanisms.

### The downregulation of MCMBP inhibits PD-L1 expression through the JAK/STAT3 signaling pathway

**Figure 10:**
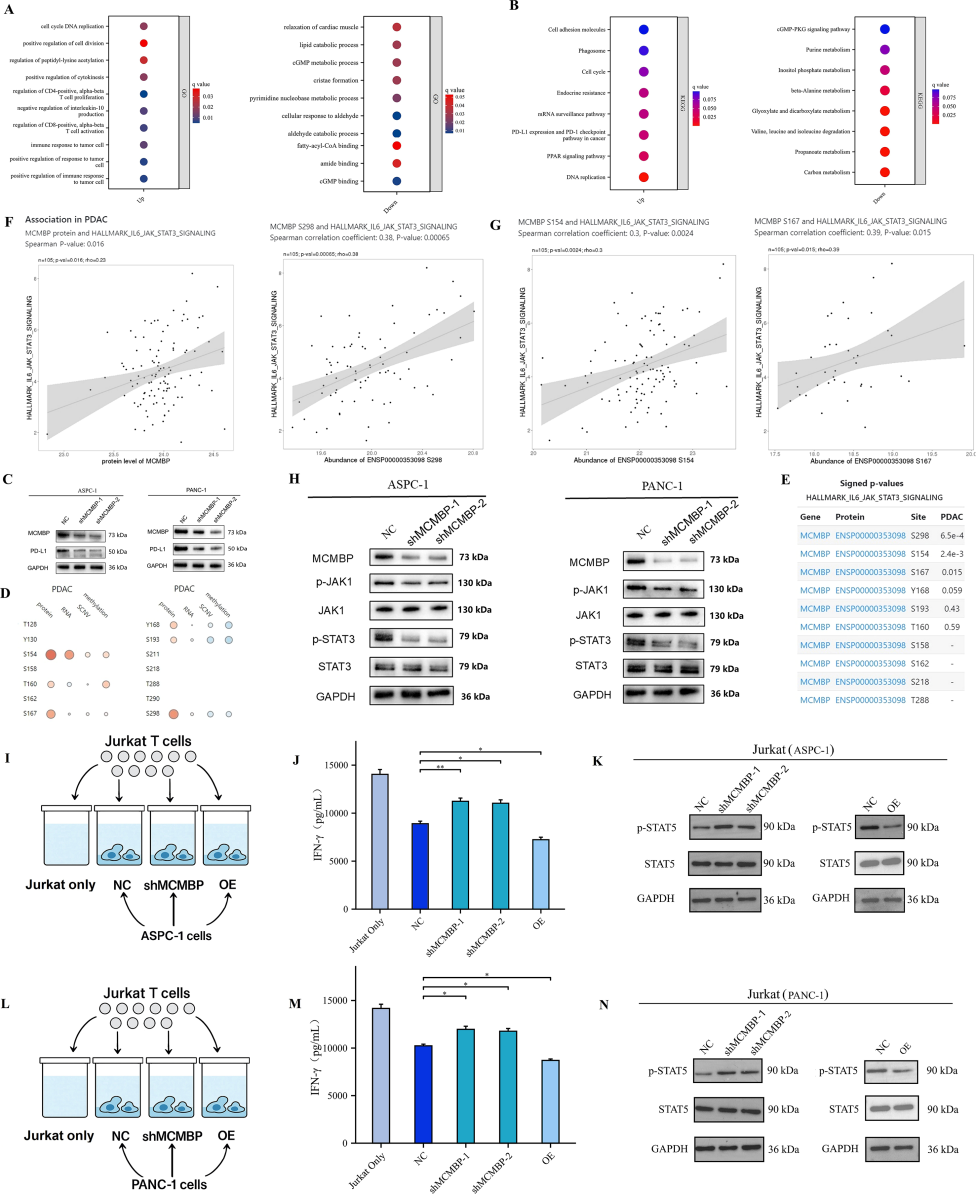
The downregulation of MCMBP inhibits PD-L1 expression through the JAK/STAT3 signaling pathway. **(A, B)** GO/KEGG pathway enrichment analysis; **(C)** PD-L1 expression in ASPC-1 and PANC-1 cell lines; **(D, E)** Correlation between MCMBP phosphorylation sites and the IL6/JAK/STAT3 signaling pathway; **(F, G)** Association of phosphorylation sites S154, S167, and S298 with the IL6/JAK/STAT3 signaling pathway; **(H)** Expression levels of P-JAK1/JAK1 and P-STAT3/STAT3 in ASPC-1 and PANC-1 cell lines; **(I)** Pattern of co-culture of control, MCMBP overexpression, and MCMBP knockdown AsPC-1 cells with Jurkat T cells; **(J)** ELISA assay for IFN-γ concentration in AsPC-1 cells; **(K)** Western blot detection of p-STAT5 and t-STAT5 protein levels in AsPC-1 cells; **(L)** Pattern of co-culture of control, MCMBP overexpression, and MCMBP knockdown PANC-1 cells with Jurkat T cells; **(M)** ELISA assay for IFN-γ concentration in PANC-1 cells; **(N)** Western blot detection of p-STAT5 and t-STAT5 protein levels in PANC-1 cells. *P < 0.05, **P < 0.01.

Analysis of the top 200 genes most correlated with MCMBP protein abundance in the TCGA-CPTAC database revealed, by GO enrichment, that these genes were primarily associated with tumor cell proliferation, migration, and immune responses upon MCMBP upregulation (**Figure 10A**). KEGG analysis further indicated enrichment of the “PD-L1 expression and PD-1 checkpoint pathway in cancer” under high MCMBP protein levels (**Figure 10B**), suggesting that MCMBP may promote immune evasion by regulating PD-L1 and correlate with poor prognosis. Western blot analysis following MCMBP knockdown in ASPC-1 and PANC-1 cells showed a decrease in PD-L1 expression (**Figure 10C**). Using the LinkedOmicsKB database, we identified significant phosphorylation changes on MCMBP at S298, S154, and S167 in PDAC, which were statistically associated with JAK-STAT3 signaling activity (**Figures 10D–G**). Subsequent Western blot experiments showed that MCMBP knockdown reduced phosphorylation levels of JAK1 and STAT3 (**Figure 10H**), indicating that the phosphorylated JAK-STAT3 pathway participates in MCMBP-mediated upregulation of PD-L1. To further validate the impact of this regulation on T cell function, we performed a direct co-culture of Jurkat T cells with control, MCMBP-overexpressing, and MCMBP-knockdown AsPC-1 and PANC-1 cells (**Figures 10I, L**). After 24 hours of CD3/CD28 activation, we assessed effector T-cell function by measuring IFN-γ secretion from Jurkat cells using ELISA. The results showed that Jurkat cells co-cultured with MCMBP-knockdown AsPC-1 cells secreted higher levels of IFN-γ than those co-cultured with control or overexpressing AsPC-1 cells (**Figure 10J**, p < 0.05). A similar trend was observed in PANC-1 cells (**Figure 10M**, p < 0.05). Furthermore, Western blot analysis of Jurkat cells harvested after co-culture showed that the phosphorylation level of STAT5 was higher in the MCMBP-knockdown group than in the control and overexpression groups in AsPC-1 cells (**Figure 10K**). A comparable change was observed in PANC-1 cells (**Figure 10N**). These results demonstrate that MCMBP knockdown in tumor cells may enhance T cell effector function. Therefore, our findings collectively indicate that MCMBP may promote immune evasion in PAAD by regulating PD-L1 expression through the JAK-STAT3 pathway and suppressing T-cell function.

## Discussion

MCMBP is a key regulator of DNA replication and cell cycle progression (9). Dysregulation of MCMBP may contribute to chromosomal instability (CIN), a recognized hallmark of tumor progression. Building on this, Quimbaya et al. demonstrated that MCMBP promotes malignant behavior and tumorigenesis in colorectal cancer, highlighting its potential as a diagnostic biomarker (12). In this study, we performed a comprehensive analysis of MCMBP expression, prognostic relevance, clinicopathological associations, epigenetic regulation, and immune interactions in PAAD using multiple public databases, with subsequent experimental validation.

Univariate and multivariate Cox regression analyses confirmed MCMBP expression to be an independent prognostic factor. To quantify its prognostic utility, survival and clinicopathological correlation analyses were conducted based on TCGA, CPTAC, and GEO datasets, leading to the development of a nomogram model for predicting patient survival probabilities.

Both DNA methylation and m6A modification play crucial regulatory roles in tumor progression, and assessing their combined effects may provide essential insights for prognostic prediction (25, 26). Our integrated analysis revealed that in PAAD, the expression of MCMBP is regulated by DNA hypomethylation and is concurrently associated with a specific m6A modification state. Notably, while our analysis did not identify a significant correlation between MCMBP expression and genetic alterations (CNV/SNV) in PAAD, its correlation with promoter hypomethylation suggests that it could be one of the key upstream factors driving MCMBP overexpression. Furthermore, high MCMBP expression correlates with elevated levels of m6A “readers” (IGF2BP1, IGF2BP3) and “writers” (VIRMA, YTHDF3), a state thought to promote tumor progression by enhancing the stability and translation of oncogenic mRNAs. Conversely, low MCMBP expression is associated with the enrichment of regulators involved in m6A demethylation (METTL16, ALKBH5, METTL14, FTO). These findings suggest that the low DNA methylation of MCMBP and its associated pro-tumorigenic m6A modification profile may synergistically contribute to the progression of PAAD prognosis.

KEGG and GO enrichment analyses implicated MCMBP in promoting PAAD cell proliferation, migration, and invasion. Moreover, MCMBP expression was positively correlated with the overall level of immune cell infiltration in the TME, specifically with the abundance of Tregs and M2 macrophages. Evaluation of 26 immune checkpoint genes further revealed that high MCMBP expression was associated with upregulation of PD-1 and PD-L1, suggesting a potential mechanism for MCMBP-mediated immune escape.

Using the TIDE algorithm, we observed that high MCMBP expression was associated with elevated TIDE, Exclusion, and PD-L1 scores, along with a lower proportion of patients responding to immunotherapy. These findings imply that elevated MCMBP expression may compromise the effectiveness of immune-based treatments. Analysis of the IMvigor210 cohort corroborated the prognostic impact of MCMBP and PD-L1 expression on patient OS, revealing a significant positive correlation between these factors. Consistently, patients with low MCMBP expression were more frequently represented among those achieving a PR or CR. Interrogation of GDSC database indicated a negative correlation between MCMBP expression and sensitivity to chemotherapeutic agents including Gemcitabine and Paclitaxel, suggesting that low MCMBP expression may predict enhanced therapeutic efficacy. Furthermore, we identified several small molecule compounds—including Tozasertib, Motesanib, AMG-232, Linifanib, and Filgotinib—that were negatively correlated with MCMBP expression and demonstrated promising therapeutic potential. Notably, pathway enrichment analysis revealed that these candidate compounds target key oncogenic pathways, including JAK-STAT and PD-L1 signaling, which are functionally aligned with MCMBP-associated processes, thereby reinforcing the potential of these drugs to counteract MCMBP-driven tumor progression. In summary, low MCMBP expression may enhance the efficacy of both immunotherapy and chemotherapy in PAAD.

In recent years, anti-PD-L1 immunotherapy has shown promising results across multiple cancer types (27–29). However, its efficacy in PAAD is often limited by an immunosuppressive TME, variable PD-L1 expression, and overactivation of inflammatory signaling pathways (30–32). Notably, aberrant activation of the JAK/STAT3 signaling pathway can upregulate PD-L1 expression and suppress T cell activity, thereby undermining the response to anti-PD-L1 therapy (33, 34). Our GSEA revealed that MCMBP-upregulated genes were significantly enriched in the JAK/STAT3 signaling pathway. To further investigate this link, we analyzed the LinkedOmicsKB database, which revealed an association between multiple MCMBP phosphorylation sites and JAK/STAT3 pathway activity. Subsequent Western blot experiments confirmed that MCMBP knockdown reduced phosphorylation of JAK1 and STAT3. Collectively, these findings suggest that These findings suggest that MCMBP may regulate PD-L1 expression through the JAK/STAT3 signaling pathway. Furthermore, our functional co-culture assays demonstrated that MCMBP-overexpressing tumor cells directly suppressed T-cell effector function, as evidenced by diminished IFN-γ secretion and reduced STAT5 phosphorylation in T cells. This indicates that MCMBP fosters an immunosuppressive microenvironment not only by upregulating PD-L1 on tumor cells but also by directly impairing T cell activation, thereby providing a more comprehensive preliminary exploration mechanistic basis for its role in immune evasion.

This study has several limitations. Firstly, the analysis predominantly relies on sample data from public databases; although we observed low methylation and high m6A modification states of MCMBP in PAAD, further experimental validation is necessary to elucidate the precise mechanisms, such as how promoter hypomethylation enhances MCMBP transcription and how the associated m6A regulators affect its mRNA stability or translation, in driving tumor progression. Secondly, due to the lack of suitable public datasets for PAAD immunotherapy, the predictive potential of MCMBP for immunotherapy response requires further validation in additional patient cohorts or preclinical models. Thirdly, the immune cell infiltration profiles, while supported by multiple computational algorithms (QUANTISEQ, ssGSEA, ImmuCellAI), remain predictions that lack direct experimental confirmation using clinical tissue samples to directly quantify differences in Tregs, CD8+ T cells, and other subsets between MCMBP high- and low-expression groups. Additionally, while our multi-omics and experimental data suggest MCMBP is an upstream regulator of the JAK-STAT3 pathway, the precise mechanism of action remains unelucidated. Whether MCMBP, as a DNA replication-related protein, regulates this signaling pathway through direct interaction or indirect means (genomic instability) remains a central question for future investigation. Lastly, the conclusions of this study are primarily based on evidence from *in vitro* experiments. While the data demonstrate the role of MCMBP in promoting malignant cell behaviors *in vitro*, its specific functions *in vivo* require further validation through animal models in future work *in vitro in vivo*.

## Conclusions

In conclusion, this study demonstrates that MCMBP holds significant prognostic value in PAAD, with its high expression closely associated with immune suppression and poor prognosis. As a biomarker related to immunotherapy, MCMBP possesses the potential to promote tumor growth and synergize with immune therapies.

## Data Availability

The original contributions presented in the study are included in the article/**Supplementary Material**. Further inquiries can be directed to the corresponding authors.
