# Supplementary figures and images for: Multiomics profiling Identifies MCMBP as a prognostic biomarker and a potential immune-related target in pancreatic ductal adenocarcinoma via the JAK–STAT3 pathway
Source: Front Immunol. 2025 Nov 19;16:1621927. doi: 10.3389/fimmu.2025.1621927 (PMC12672452; doi:10.3389/fimmu.2025.1621927)

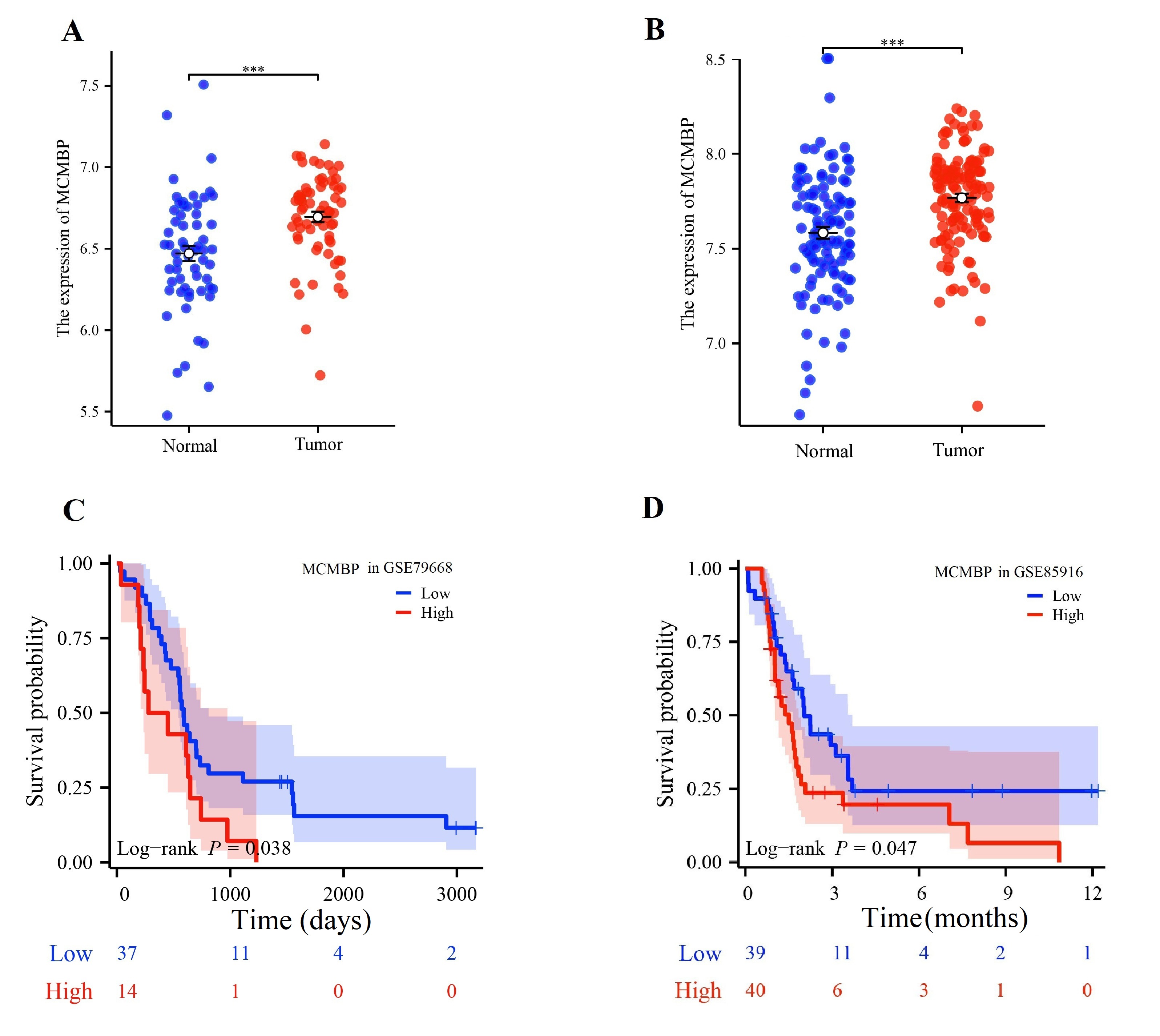

Supplement: Supplementary file 1 [file Image1.jpeg]

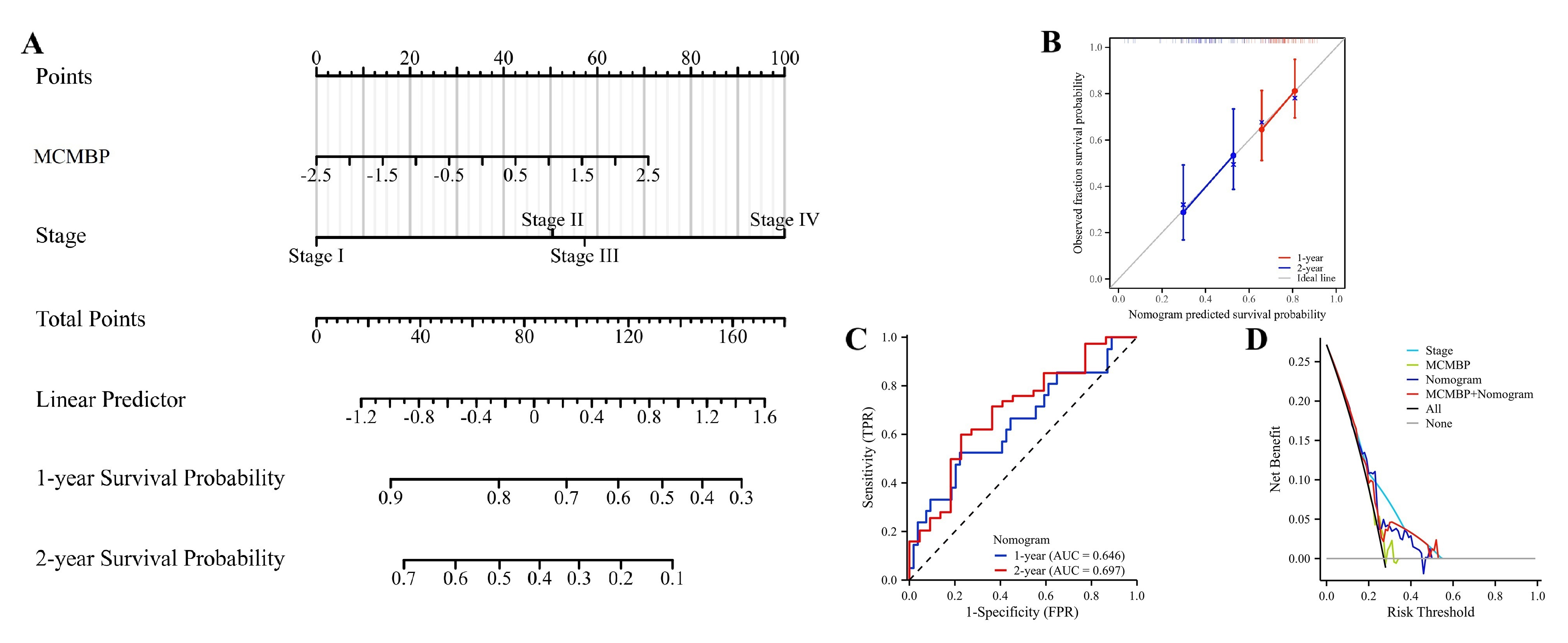

Supplement: Supplementary file 2 [file Image2.jpeg]

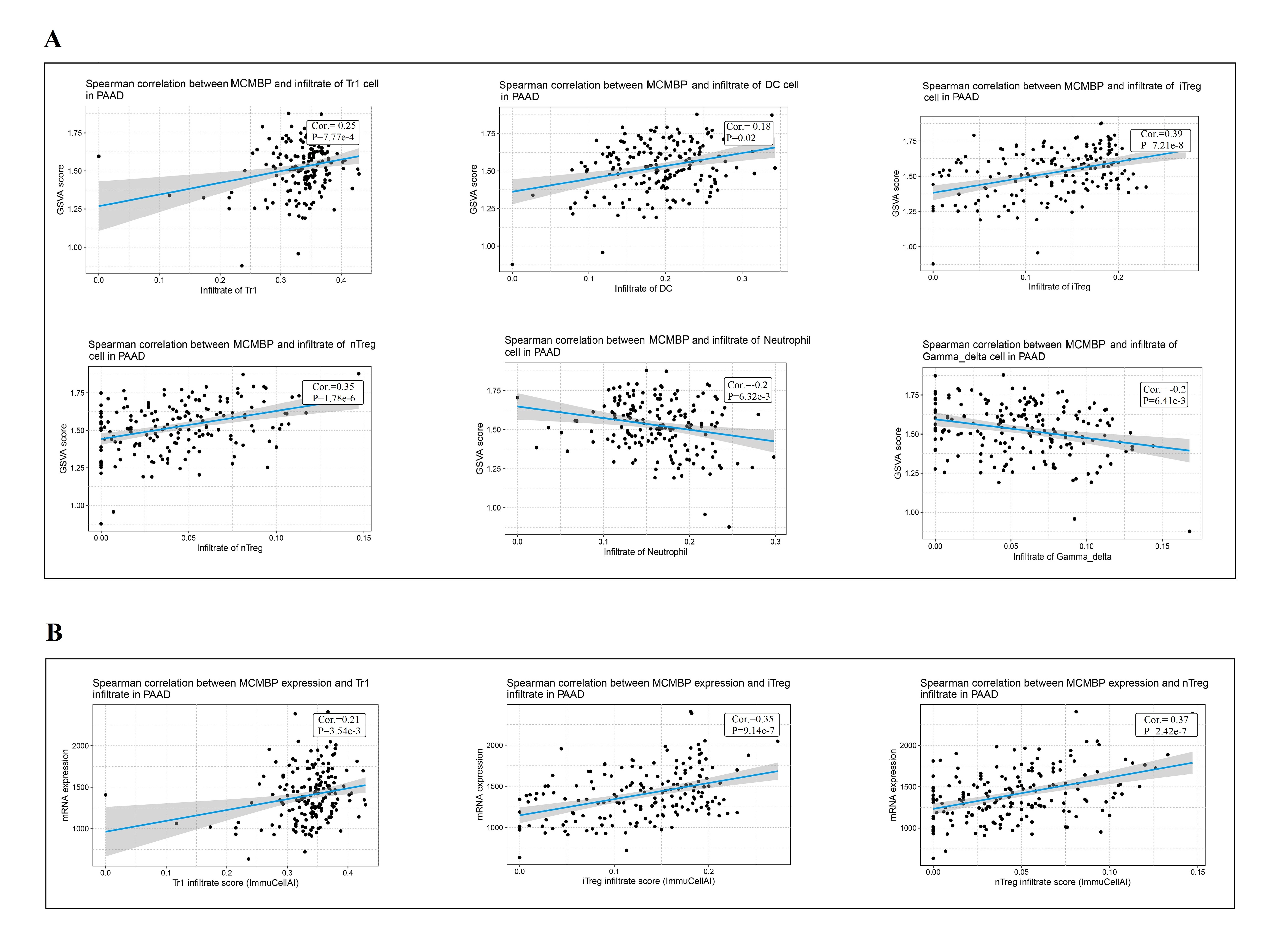

Supplement: Supplementary file 3 [file Image3.jpeg]

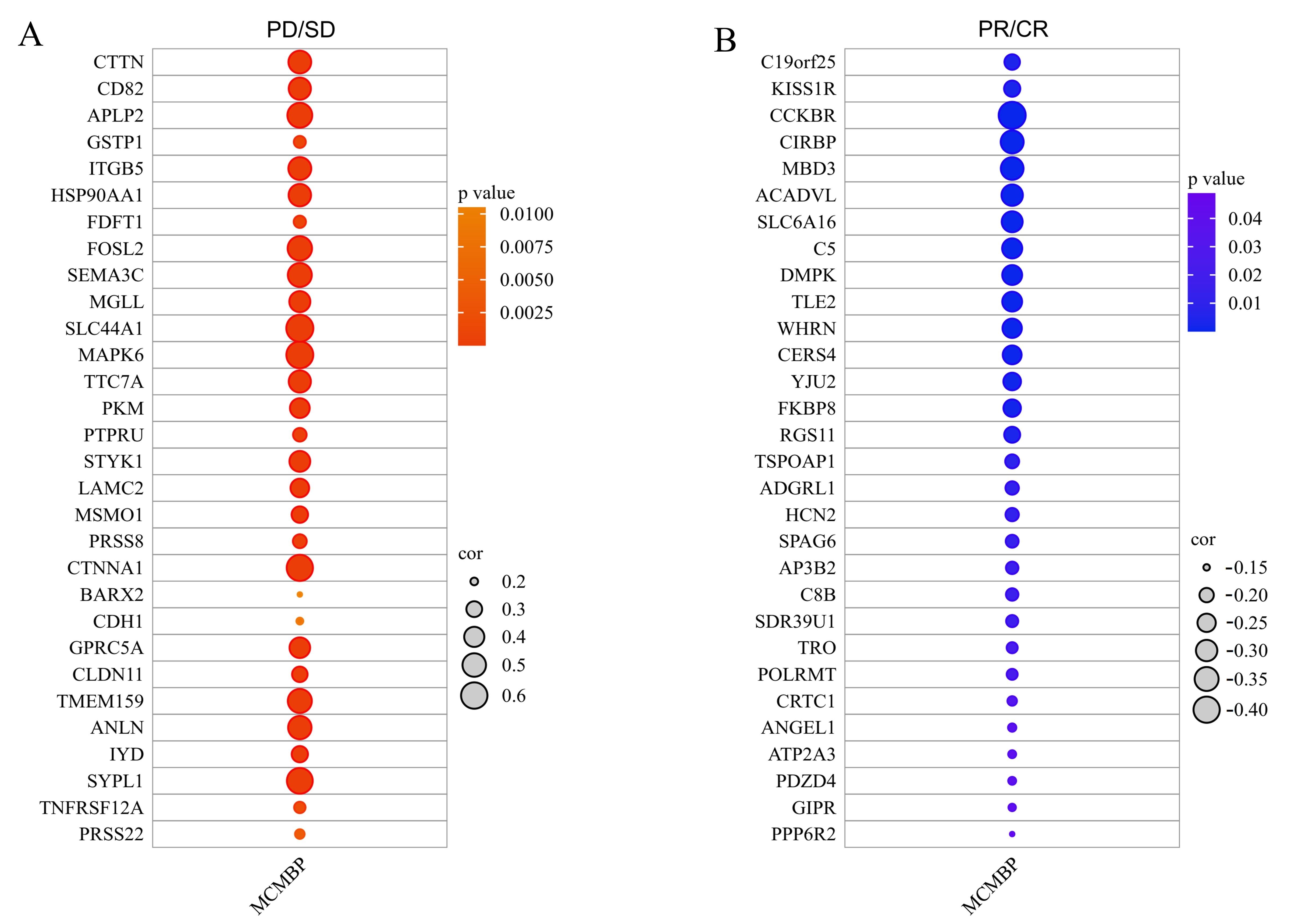

Supplement: Supplementary file 4 [file Image4.jpeg]
